# Older Patients With Memory Complaints Often Prefer Diagnostics in Primary Care or No Further Diagnostic Evaluation

**DOI:** 10.1002/gps.70229

**Published:** 2026-06-16

**Authors:** Demi Ronner, Edo Richard, Eric P. Moll van Charante, Willem A. van Gool, Henk J. Schers, Marieke Perry

**Affiliations:** ^1^ Department of Primary and Community Care Radboud University Medical Center Radboudumc Alzheimer Centre Nijmegen the Netherlands; ^2^ Department of Neurology Donders Institute for Brain, Cognition, and Behaviour Radboud University Medical Center Nijmegen the Netherlands; ^3^ Department of Public and Occupational Health Amsterdam University Medical Centre University of Amsterdam Amsterdam the Netherlands; ^4^ Department of General Practice Amsterdam University Medical Centre University of Amsterdam Amsterdam the Netherlands

## Conflicts of Interest

The authors declare no conflicts of interest.

Dementia can be diagnosed in primary care or in memory clinics (MCs). General practitioners (GPs) may offer less burdensome diagnostic trajectories, including inconclusive wait‐and‐see strategies [1]. MCs may provide faster and more comprehensive diagnostics [2], but at a higher cost and with a risk of overdiagnosis or incidental findings [3, 4]. Dutch guidelines support both pathways, depending on clinical presentation and patient preferences. Although research agendas suggest MC superiority, this was never investigated with regard to patient‐relevant outcomes [5].

We initiated the PRImary care and MEmory clinic Diagnostics (PRIMED) trial, a randomised non‐inferiority study comparing primary care– versus MC‐based dementia diagnostics on long‐term patient‐ and caregiver‐relevant outcomes and costs [6]. Trial recruitment progressed more slowly than anticipated. Our aim was to investigate why patients declined participation and why healthcare professionals refrained from inviting them to participate.

We performed an exploratory analysis using data from twenty participating Dutch general practices during the first 6 months of PRIMED. Patients aged ≥ 70 years with memory complaints were identified by searching electronic medical health records using the International Classification of Primary Care (ICPC) code P20 (memory disturbances). We recorded invitation and participation status and documented reasons for non‐invitation or declining participation.

**FIGURE 1:**
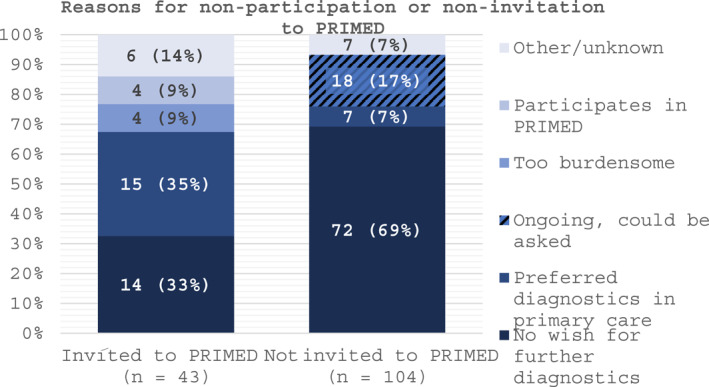
100%‐stacked bar chart presenting patients' reasons for (non‐)participation and healthcare professionals' reasons for not inviting patients to PRIMED. Values are presented as *n* (%).

We identified 219 patients aged ≥ 70 years with ICPC code P20, of whom 199 presented with memory complaints. 52 patients were not eligible for PRIMED participation: 37 had already been referred to an MC or had received a diagnosis at the time of identification, eight had a terminal illness or were considered too frail, six had an indication for referral to an MC, and one could not participate due to a language barrier. The 147 patients who were eligible had a mean age of 82.7 years (SD 6.0), and 61.4% were women. Only 43 patients (29%) were invited to participate in PRIMED, 4 (9%) of whom agreed (Figure 1). Fifteen patients (35%) declined because they preferred diagnostic evaluation in primary care, fourteen (33%) did not want further diagnostics at that time, four (9%) found participation too burdensome, and six (14%) declined for other reasons. No patient declined because of a preference for MC referral.

Among the 104 eligible patients who were not invited, healthcare professionals reported that 72 (69%) did not wish to undergo further diagnostic evaluation, seven (7%) preferred to remain in primary care for diagnostics, eighteen (17%) had not been invited but could still be approached, and seven (7%) were not invited for other reasons (Figure 1).

Our findings show that many Dutch older patients with memory complaints prefer either diagnostic assessment in primary care or no further diagnostics at all. One in three patients who declined participation explicitly preferred remaining in primary care, and only a small minority consented to randomisation between diagnostic pathways.

Our results challenge the assumption—implicit in many guidelines and research agendas—that early, specialist‐led dementia diagnosis is universally desired. Instead, they support the concept of a ‘timely’ diagnosis, aligned with individual goals, preferences, and circumstances [7]. In everyday practice, GPs often encounter patients who are ambivalent about diagnostic testing or fear that a diagnosis may lead to anxiety, reduced quality of life, or premature institutionalisation [8]. Navigating these concerns requires balancing patient autonomy with non‐maleficence and may legitimately result in postponing or avoiding specialist referral [9].

Our findings confirm the difficulty of recruiting older patients into randomised trials conducted in primary care [10]. Among eligible patients, invitation rates were low, and actual participation was minimal. These findings raise questions about the feasibility of randomised designs that assume willingness to undergo diagnostic evaluation and random allocation between care pathways, an approach that excludes patient preferences from diagnostic decision‐making.

Although reasons for non‐invitation were based on healthcare professionals' retrospective reports—and may therefore reflect clinical judgement rather than patients' explicitly stated preferences—the overall pattern indicates limited enthusiasm for MC referral among many patients. Future qualitative research could provide insight into patient preferences for primary care diagnostics and avoidance of further diagnostic evaluation.

In conclusion, we observed a clear mismatch between the preferences of many older patients for primary care–based assessment or no further diagnostics and the research emphasis on early, specialist‐led dementia diagnosis. Incorporating patient preferences into trial design may be essential for successful recruitment. More flexible diagnostic strategies that take these preferences into account are also crucial for delivering patient‐centred dementia care.

## Funding

This work was supported by the ZonMw (10390012110040).

## Data Availability

The data that support the findings of this study are available from the corresponding author upon reasonable request.
